# Supplementary material for: Overall time spent by clients from entry to exit and associated factors in out-patient departments in public hospitals of Jimma Zone southwest, Ethiopia
Source: PLoS One. 2024 Mar 7;19(3):e0296630. doi: 10.1371/journal.pone.0296630 (PMC10919670; doi:10.1371/journal.pone.0296630)
Supplement: S1 Table — A. Waiting time (in a minute) at each section of OPD in Jimma zone public hospitals 2018.(n = 236). B. The total waiting time the patient spends in OPD of Jimma zone public hospitals 2018. (236). (ZIP) [file pone.0296630.s001.zip › SI S1A table.docx]

| **S1A Table : waiting time (in minute) at each section of OPD in Jimma zone public hospitals 2018.(n=236)** | | | | | | | | | | |
| --- | --- | --- | --- | --- | --- | --- | --- | --- | --- | --- |
| Name of the hospital | | Registration | Triage | Examination(OPD) | Laboratory | | X-ray | | Other Dixcs*** | Pharmacy |
|  |  |  |  |  | Pre | Post | Pre | Post |  |  |
| JUMC | Mean | 20.59 | 23.75 | 67.86 | 71.26 | 103.77 | 78.64 | 11.00 | 67.62 | 6.17 |
|  | Median | 10.00 | 20.00 | 61.00 | 58.00 | 96.00 | 78.00 | 3.00 | 51.00 | 5.00 |
|  | Minimum | ** | 2 | 1 | 10 | 38 | 15 | 1 | 2 | ** |
|  | Maximum | 246 | 130 | 300 | 205 | 290 | 158 | 81 | 333 | 57 |
|  | Std. Deviation | 27.981 | 22.139 | 43.936 | 40.450 | 44.943 | 46.728 | 21.170 | 72.914 | 6.512 |
| Agaro general hospital | Mean | 17.24 | 13.53 | 59.39 | 51.94 | 97.56 | 80.00 | 12.50 | 66.00 | 7.29 |
|  | Median | 11.50 | 13.00 | 54.50 | 46.00 | 88.50 | 63.00 | 3.00 | 50.00 | 6.00 |
|  | Minimum | 1 | 2 | 1 | 10 | 27 | 31 | 1 | 34 | ** |
|  | Maximum | 90 | 30 | 265 | 131 | 166 | 163 | 43 | 130 | 54 |
|  | Std. Deviation | 17.922 | 7.392 | 45.053 | 33.980 | 45.999 | 57.498 | 20.421 | 43.451 | 9.164 |
| Seka primary hospital | Mean | 11.24 | 20.11 | 38.67 | 69.00 | 83.75 | 76.00 | 10.00 | 36.00 | 4.27 |
|  | Median | 8.00 | 16.00 | 33.00 | 56.50 | 91.50 | 76.00 | 10.00 | 48.00 | 3.00 |
|  | Minimum | 1 | 4 | 1 | 37 | 39 | 76 | 10 | 5 | ** |
|  | Maximum | 40 | 53 | 96 | 126 | 113 | 76 | 10 | 55 | 15 |
|  | Std. Deviation | 10.981 | 15.664 | 29.648 | 39.573 | 35.678 | . | . | 27.074 | 4.166 |
| Total | Mean | 19.22 | 22.19 | 63.90 | 68.64 | 102.36 | 78.79 | 11.26 | 64.00 | 6.22 |
|  | Median | 10.00 | 18.00 | 58.00 | 56.00 | 95.00 | 68.00 | 3.00 | 49.50 | 5.00 |
|  | Minimum | ** | 2 | 1 | 10 | 27 | 15 | 1 | 2 | ** |
|  | Maximum | 246 | 130 | 300 | 205 | 290 | 163 | 81 | 333 | 57 |
|  | Std. Deviation | 25.592 | 20.653 | 43.728 | 39.909 | 44.727 | 46.138 | 19.841 | 65.575 | 6.895 |

^**presents zero waiting time (after arrived immediately gained the services)^

^*** presents ultrasound, sputum examination & FNA/C^
