# Supplementary material for: Overall time spent by clients from entry to exit and associated factors in out-patient departments in public hospitals of Jimma Zone southwest, Ethiopia
Source: PLoS One. 2024 Mar 7;19(3):e0296630. doi: 10.1371/journal.pone.0296630 (PMC10919670; doi:10.1371/journal.pone.0296630)
Supplement: S1 Table — A. Waiting time (in a minute) at each section of OPD in Jimma zone public hospitals 2018.(n = 236). B. The total waiting time the patient spends in OPD of Jimma zone public hospitals 2018. (236). (ZIP) [file pone.0296630.s001.zip › SI S1B table.docx]

**S1B table : the total waiting time the patient spends in OPD of Jimma zone public hospitals 2018. (236)**

| Name of the hospital | Mean | Median | Minimum | Maximum | Std. Deviation |
| --- | --- | --- | --- | --- | --- |
| JUMC | 235.40 | 238.00 | 43 | 557 | 117.563 |
| Agaro general hospital | 176.76 | 180.00 | 13 | 378 | 114.274 |
| Seka primary hospital | 99.90 | 71.00 | 5 | 370 | 97.362 |
| Total | 213.90 | 222.50 | 5 | 557 | 122.255 |
