# Supplementary material for: Overall time spent by clients from entry to exit and associated factors in out-patient departments in public hospitals of Jimma Zone southwest, Ethiopia
Source: PLoS One. 2024 Mar 7;19(3):e0296630. doi: 10.1371/journal.pone.0296630 (PMC10919670; doi:10.1371/journal.pone.0296630)
Supplement: S2 Table — A. Service times (minutes) within the different sections of OPD in Jimma zone public hospitals 2018. (n = 236). B. The service time in minutes based on the type of OPD at Jimma zone public hospitals 2018.(n = 236). C. The total service time the patient spends in OPD of Jimma zone public hospitals 2018. (n = 236). (ZIP) [file pone.0296630.s002.zip › SI S2C table.docx]

**S2 C table: the total service time the patient spends in OPD of Jimma zone public hospitals 2018. (n=236)**

| Name of hospital | Mean | Median | Minimum | Maximum | Std. Deviation |
| --- | --- | --- | --- | --- | --- |
| JUMC | 50.01 | 43.00 | 21 | 279 | 29.609 |
| Agaro general hospital | 55.37 | 47.50 | 24 | 155 | 26.798 |
| Seka primary hospital | 43.90 | 40.00 | 9 | 95 | 19.736 |
| Total | 50.33 | 43.50 | 9 | 279 | 28.473 |
