# Supplementary material for: Overall time spent by clients from entry to exit and associated factors in out-patient departments in public hospitals of Jimma Zone southwest, Ethiopia
Source: PLoS One. 2024 Mar 7;19(3):e0296630. doi: 10.1371/journal.pone.0296630 (PMC10919670; doi:10.1371/journal.pone.0296630)
Supplement: S2 Table — A. Service times (minutes) within the different sections of OPD in Jimma zone public hospitals 2018. (n = 236). B. The service time in minutes based on the type of OPD at Jimma zone public hospitals 2018.(n = 236). C. The total service time the patient spends in OPD of Jimma zone public hospitals 2018. (n = 236). (ZIP) [file pone.0296630.s002.zip › SI S2A table.docx]

**S2A table : service times (minutes) with in different section of OPD in Jimma zone public hospitals 2018. (n=236)**

| Name of the hospital | | Registration | Examination (OPD) | Laboratory | x-ray | Other Dixcs*** | pharmacy |
| --- | --- | --- | --- | --- | --- | --- | --- |
| JUMC | Mean | 9.34 | 23.98 | 5.30 | 9.00 | 56.89 | 8.63 |
|  | Median | 8.00 | 22.00 | 5.00 | 9.00 | 30.00 | 7.00 |
|  | Minimum | 2 | 7 | 2 | 7 | 10 | 2 |
|  | Maximum | 55 | 105 | 13 | 11 | 210 | 32 |
|  | Std. Deviation | 6.270 | 13.309 | 2.707 | 1.177 | 58.533 | 5.436 |
| Agaro general hospital | Mean | 10.55 | 30.42 | 5.72 | 10.00 | 31.00 | 9.15 |
|  | Median | 10.00 | 24.50 | 5.00 | 9.50 | 32.00 | 7.00 |
|  | Minimum | 2 | 8 | 2 | 9 | 29 | 3 |
|  | Maximum | 40 | 100 | 17 | 12 | 32 | 32 |
|  | Std. Deviation | 7.439 | 20.014 | 3.545 | 1.414 | 1.732 | 5.955 |
| Seka primary hospital | Mean | 8.33 | 25.81 | 5.25 | 9.00 | 25.67 | 6.53 |
|  | Median | 7.00 | 22.00 | 4.50 | 9.00 | 25.00 | 6.00 |
|  | Minimum | 2 | 4 | 4 | 9 | 12 | 2 |
|  | Maximum | 20 | 63 | 8 | 9 | 40 | 14 |
|  | Std. Deviation | 5.013 | 15.760 | 1.893 | . | 14.012 | 3.091 |
| Total | Mean | 9.45 | 25.19 | 5.35 | 9.21 | 50.04 | 8.56 |
|  | Median | 8.00 | 22.00 | 5.00 | 9.00 | 30.00 | 7.00 |
|  | Minimum | 2 | 4 | 2 | 7 | 10 | 2 |
|  | Maximum | 55 | 105 | 17 | 12 | 210 | 32 |
|  | Std. Deviation | 6.373 | 14.920 | 2.796 | 1.228 | 52.373 | 5.403 |

^*** presents ultrasound, sputum examination & FNA/C^
