# Supplementary material for: Overall time spent by clients from entry to exit and associated factors in out-patient departments in public hospitals of Jimma Zone southwest, Ethiopia
Source: PLoS One. 2024 Mar 7;19(3):e0296630. doi: 10.1371/journal.pone.0296630 (PMC10919670; doi:10.1371/journal.pone.0296630)
Supplement: S2 Table — A. Service times (minutes) within the different sections of OPD in Jimma zone public hospitals 2018. (n = 236). B. The service time in minutes based on the type of OPD at Jimma zone public hospitals 2018.(n = 236). C. The total service time the patient spends in OPD of Jimma zone public hospitals 2018. (n = 236). (ZIP) [file pone.0296630.s002.zip › SI S2B table.docx]

**S2B table: the service time in minutes based on type of OPD at Jimma zone public hospitals 2018.(n=236)**

| Name of the hospital | after triage/registration to what unit patient go | | Registration | Examination (OPD) | Laboratory | x-ray | Other Dixcs*** units | pharmacy |
| --- | --- | --- | --- | --- | --- | --- | --- | --- |
| JUMC | medical | Mean | 9.81 | 23.91 | 5.68 | 9.00 | 74.33 | 8.98 |
|  |  | Median | 8.00 | 22.00 | 5.00 | 9.00 | 48.00 | 7.00 |
|  |  | Minimum | 2 | 8 | 2 | 8 | 28 | 2 |
|  |  | Maximum | 55 | 89 | 13 | 11 | 170 | 32 |
|  |  | Std. Deviation | 7.115 | 12.068 | 2.839 | 1.069 | 54.277 | 6.082 |
|  | surgical | Mean | 8.97 | 22.70 | 4.44 | 9.00 | 47.88 | 7.69 |
|  |  | Median | 8.50 | 22.00 | 4.00 | 9.00 | 23.50 | 7.00 |
|  |  | Minimum | 2 | 10 | 2 | 7 | 13 | 3 |
|  |  | Maximum | 20 | 50 | 9 | 11 | 210 | 19 |
|  |  | Std. Deviation | 3.810 | 9.958 | 2.063 | 1.414 | 66.632 | 3.646 |
|  | gyn | Mean | 8.18 | 28.45 | 5.78 |  | 14.50 | 8.50 |
|  |  | Median | 6.00 | 15.00 | 6.00 |  | 14.50 | 7.50 |
|  |  | Minimum | 2 | 10 | 2 |  | 10 | 5 |
|  |  | Maximum | 17 | 105 | 10 |  | 19 | 15 |
|  |  | Std. Deviation | 4.665 | 27.226 | 3.032 |  | 6.364 | 3.854 |
|  | ophta | Mean | 5.71 | 17.29 | 2.50 |  |  | 7.14 |
|  |  | Median | 6.00 | 15.00 | 2.50 |  |  | 7.00 |
|  |  | Minimum | 3 | 10 | 2 |  |  | 5 |
|  |  | Maximum | 8 | 27 | 3 |  |  | 10 |
|  |  | Std. Deviation | 1.604 | 6.775 | .707 |  |  | 1.952 |
|  | dental | Mean | 7.75 | 37.75 |  |  |  | 15.50 |
|  |  | Median | 7.50 | 38.50 |  |  |  | 15.50 |
|  |  | Minimum | 4 | 23 |  |  |  | 11 |
|  |  | Maximum | 12 | 51 |  |  |  | 20 |
|  |  | Std. Deviation | 3.862 | 14.818 |  |  |  | 6.364 |
|  | mental | Mean | 10.00 | 22.25 | 3.50 |  |  | 6.00 |
|  |  | Median | 7.50 | 15.00 | 3.50 |  |  | 6.00 |
|  |  | Minimum | 5 | 7 | 3 |  |  | 4 |
|  |  | Maximum | 20 | 52 | 4 |  |  | 8 |
|  |  | Std. Deviation | 6.880 | 20.205 | .707 |  |  | 1.826 |
| Agaro general hospital | medical | Mean | 10.57 | 31.00 | 6.64 | 10.33 | 30.50 | 9.52 |
|  |  | Median | 10.00 | 24.00 | 6.00 | 10.00 | 30.50 | 7.00 |
|  |  | Minimum | 2 | 8 | 3 | 9 | 29 | 3 |
|  |  | Maximum | 40 | 100 | 17 | 12 | 32 | 32 |
|  |  | Std. Deviation | 7.375 | 21.973 | 4.056 | 1.528 | 2.121 | 6.571 |
|  | surgical | Mean | 7.80 | 22.00 | 4.25 | 9.00 |  | 7.75 |
|  |  | Median | 8.00 | 22.00 | 4.00 | 9.00 |  | 8.00 |
|  |  | Minimum | 5 | 18 | 2 | 9 |  | 5 |
|  |  | Maximum | 11 | 26 | 7 | 9 |  | 10 |
|  |  | Std. Deviation | 2.387 | 3.162 | 2.217 | . |  | 2.630 |
|  | gyn | Mean | 15.00 | 38.67 | 4.33 |  | 32.00 | 7.67 |
|  |  | Median | 10.00 | 36.00 | 3.00 |  | 32.00 | 7.00 |
|  |  | Minimum | 5 | 30 | 3 |  | 32 | 6 |
|  |  | Maximum | 30 | 50 | 7 |  | 32 | 10 |
|  |  | Std. Deviation | 13.229 | 10.263 | 2.309 |  | . | 2.082 |
| Seka primary hospital | medical | Mean | 8.76 | 25.18 | 6.00 | 9.00 | 40.00 | 6.69 |
|  |  | Median | 7.00 | 25.00 | 6.00 | 9.00 | 40.00 | 6.00 |
|  |  | Minimum | 2 | 4 | 4 | 9 | 40 | 2 |
|  |  | Maximum | 20 | 60 | 8 | 9 | 40 | 14 |
|  |  | Std. Deviation | 5.460 | 14.336 | 2.828 | . | . | 3.301 |
|  | surgical | Mean | 6.00 | 34.33 | 5.00 |  | 18.50 | 6.00 |
|  |  | Median | 5.00 | 21.00 | 5.00 |  | 18.50 | 6.00 |
|  |  | Minimum | 5 | 19 | 5 |  | 12 | 6 |
|  |  | Maximum | 8 | 63 | 5 |  | 25 | 6 |
|  |  | Std. Deviation | 1.732 | 24.846 | . |  | 9.192 | . |
|  | gyn | Mean | 8.00 | 11.00 | 4.00 |  |  | 5.00 |
|  |  | Median | 8.00 | 11.00 | 4.00 |  |  | 5.00 |
|  |  | Minimum | 8 | 11 | 4 |  |  | 5 |
|  |  | Maximum | 8 | 11 | 4 |  |  | 5 |
|  |  | Std. Deviation | . | . | . |  |  | . |
| Total | medical | Mean | 9.84 | 25.34 | 5.80 | 9.33 | 64.17 | 8.87 |
|  |  | Median | 8.00 | 23.00 | 5.00 | 9.00 | 36.00 | 7.00 |
|  |  | Minimum | 2 | 4 | 2 | 8 | 28 | 2 |
|  |  | Maximum | 55 | 100 | 17 | 12 | 170 | 32 |
|  |  | Std. Deviation | 6.989 | 14.733 | 2.992 | 1.231 | 49.867 | 5.993 |
|  | surgical | Mean | 8.62 | 23.46 | 4.44 | 9.00 | 42.00 | 7.65 |
|  |  | Median | 8.00 | 22.00 | 4.00 | 9.00 | 22.50 | 7.00 |
|  |  | Minimum | 2 | 10 | 2 | 7 | 12 | 3 |
|  |  | Maximum | 20 | 63 | 9 | 11 | 210 | 19 |
|  |  | Std. Deviation | 3.615 | 10.991 | 2.015 | 1.291 | 60.133 | 3.463 |
|  | gyn | Mean | 9.53 | 29.33 | 5.31 |  | 20.33 | 8.00 |
|  |  | Median | 8.00 | 20.00 | 4.00 |  | 19.00 | 6.50 |
|  |  | Minimum | 2 | 10 | 2 |  | 10 | 5 |
|  |  | Maximum | 30 | 105 | 10 |  | 32 | 15 |
|  |  | Std. Deviation | 6.968 | 24.245 | 2.750 |  | 11.060 | 3.357 |
|  | ophta | Mean | 5.71 | 17.29 | 2.50 |  |  | 7.14 |
|  |  | Median | 6.00 | 15.00 | 2.50 |  |  | 7.00 |
|  |  | Minimum | 3 | 10 | 2 |  |  | 5 |
|  |  | Maximum | 8 | 27 | 3 |  |  | 10 |
|  |  | Std. Deviation | 1.604 | 6.775 | .707 |  |  | 1.952 |
|  | dental | Mean | 7.75 | 37.75 |  |  |  | 15.50 |
|  |  | Median | 7.50 | 38.50 |  |  |  | 15.50 |
|  |  | Minimum | 4 | 23 |  |  |  | 11 |
|  |  | Maximum | 12 | 51 |  |  |  | 20 |
|  |  | Std. Deviation | 3.862 | 14.818 |  |  |  | 6.364 |
|  | mental | Mean | 10.00 | 22.25 | 3.50 |  |  | 6.00 |
|  |  | Median | 7.50 | 15.00 | 3.50 |  |  | 6.00 |
|  |  | Minimum | 5 | 7 | 3 |  |  | 4 |
|  |  | Maximum | 20 | 52 | 4 |  |  | 8 |
|  |  | Std. Deviation | 6.880 | 20.205 | .707 |  |  | 1.826 |
