# Supplementary material for: Overall time spent by clients from entry to exit and associated factors in out-patient departments in public hospitals of Jimma Zone southwest, Ethiopia
Source: PLoS One. 2024 Mar 7;19(3):e0296630. doi: 10.1371/journal.pone.0296630 (PMC10919670; doi:10.1371/journal.pone.0296630)
Supplement: S3 Table — (DOCX) [file pone.0296630.s003.docx]

**S3 table**: **overall time patient spent from entrey to exit in OPD of Jimma zone public hospitals 2018. (n=236)**

| Name of the hospital | Mean | Median | Minimum | Maximum | Std. Deviation |
| --- | --- | --- | --- | --- | --- |
| JUMC | 336.64 | 359.00 | 86 | 1180 | 158.726 |
| Agaro general hospital | 275.58 | 303.00 | 59 | 490 | 144.089 |
| Seka primary hospital | 173.86 | 148.00 | 37 | 483 | 115.757 |
| Total | 312.33 | 342.50 | 37 | 1180 | 160.172 |
