# Supplementary material for: Overall time spent by clients from entry to exit and associated factors in out-patient departments in public hospitals of Jimma Zone southwest, Ethiopia
Source: PLoS One. 2024 Mar 7;19(3):e0296630. doi: 10.1371/journal.pone.0296630 (PMC10919670; doi:10.1371/journal.pone.0296630)
Supplement: Attachment — Submitted filename: Response to Reviewer.docx [file pone.0296630.s006.docx]

Date: December 5, 2022

Manuscript ID: PONE-D-22-02050

Overall Time spent by clients from entry to exit and associated factors in out-patient departments in public hospitals of Jimma Zone Southwest, Ethiopia

Corresponding authors: Zebader Walle (MPh)

Dear Dr. Dragan Pamucar

PLOS ONE

Thank you for your letter dated September 5, 2022 with a decision of major revision needed. We were pleased to know that our manuscript was considered potentially acceptable for publication in PLoS ONE, subject to adequate revision as requested by the reviewers, academic editors and the journal requirements. Based on the instructions provided in your letter, we uploaded the file of the rebuttal letter; the marked up copy of the revised manuscript highlighting the changes made in the original submitted version and the clean copy of the revised manuscript.

We have revised all sections of the manuscript using the comments from the editors, independent reviewers, and the standard guideline of the journal. Hence, we have marked in red color all the changes made during the revision process. Beside this letter is our point-by-point response (rebuttal letter) to the comments made by the reviewers and a minimal data set is prepared in supporting information in the revised manuscript. We have agreed with almost all the comments and questions raised by the reviewers and academic editors justification were provided for the disagreed comments and questions accordingly. We would like to take this opportunity to express our thanks to the reviewers for their valuable comments and to thank you for allowing us to resubmit a revision of the manuscript. The authors have declared that no competing interests exist.

I hope that the revised manuscript is accepted for publication in PLoS ONE.

Sincerely yours,

Zebader Walle (Debre Tabor University)

**Response to editor**

**Question: #1.** The abstract is loosely written. It is not as informative as expected. A standard abstract must present, without leaving any doubt, the objective of the paper precisely; source of data (which is not present in your abstract) and analytical approach used; key findings and any policy implication and recommendations.

**Response: thank you very much for your critical comments. We have modified all the raised issues during the revision process (please see the revised version of the manuscript)**

**Question: #2.** - I suggest the authors to improve the introduction section. Authors should better highlight the objective of their work and to what extent it contributes to close a gap in the existing literature and/or practice. What is the innovative value of the contribution proposed by the authors?

**Response: thank you for this remark. Even though, some papers done focusing on a single unit waiting time the overall time in terms of waiting and service time the patient spent in healthcare facilities was not studied but this study aims to address thus issues.**

**Question: #3.** You should provide more recent references published in last two-three years in the Literature review. Remove references published before 2018.

**Response: thank you for your comment. There is no enough papers within two-three years that talk about the time patient spent in hospitals.**

**Question: #4.** Explain in more details in the data used in the case study, the data for the testing, the criterion for the accuracy, and others to claim these points.

**Response: thank you for your constructive comments. We have corrected it on the revised manuscript based on the request.**

**Question: #5.** Discussion section. How should we know about the quality of these solutions? Could you compare these results with some existing approaches in literature? The improvement must be discussed.

**Response**: **thank you for your comment. We have try to discussed the result with literatures and the Jimma zone hospital approach (citizen charter).**

**Questions: #6.** The conclusion section seems to rush to the end. The authors will have to demonstrate the impact and insights of the research. The authors need to clearly provide several solid future research directions. Clearly state your unique research contributions in the conclusion section. Add limitations of the model. No bullets should be used in your conclusion section.

**Response:** Thank you for your remark. We have modified it in the revised manuscript.

**Question: #7.** Please ensure that your manuscript meets PLOS ONE's style requirements, including those for file naming. The PLOS ONE style templates can be found at

<https://journals.plos.org/plosone/s/file?id=wjVg/PLOSOneformatting_sample_main_body.pdf> and

<https://journals.plos.org/plosone/s/file?id=ba62/PLOSOne_formatting_sample_title_authors_affiliations.pdf>

**Response: Thank you for this remark. We re-formatted the revised manuscript using the PLoS ONE format guidelines including naming of the file. The whole content of the manuscript, including the abstract, introduction, methods, discussion and reference are formatted using the guidelines of the journal (Please see the revised version of the manuscript).**

**Question: #8.** You indicated that you had ethical approval for your study. In your Methods section, please ensure you have also stated whether you obtained consent from parents or guardians of the minors included in the study or whether the research ethics committee or IRB specifically waived the need for their consent.

**Response: thank you for your comment. We have set the required information on the Ethical consideration (method) part so you can see it on the revised manuscript.**

**Question: #9.** In the ethics statement in the Methods, you have specified that verbal consent was obtained. Please provide additional details regarding how this consent was documented and witnessed, and state whether this was approved by the IRB"

**Response:** thank you for your insight. Because the study participants have different backgrounds such as educational background that make them fear of to take written consent (especially unable to read and write individuals were unable to take it). We have obtained approval from IRB of institute of health science Jimma University we performed the study.

**Question: #10.** . Please include additional information regarding the survey or questionnaire used in the study and ensure that you have provided sufficient details that others could replicate the analyses. For instance, if you developed a questionnaire as part of this study and it is not under a copyright more restrictive than CC-BY, please include a copy, in both the original language and English, as Supporting Information.""

**Response: thank you for your concern. The questionnaire was adapt by reviewing different literatures, for more information all things regarding to the tool incorporates in the method part of the revised manuscript (see it).**

**Question: #11.** We note that you included minors (age<18) in your study. Please provide additional details regarding minors consent. In the ethics statement in the Methods and online submission information, please ensure that you have specified whether you obtained consent from parents or guardians. If the need for consent was waived by the ethics committee, please include this information.

**Response**: thank you for your comment. We have get the consent from their parents/ relatives who came with them(for more information you can see the revised manuscript).

**Question: #12.** Thank you for stating the following in the Acknowledgments Section of your manuscript:

“We would like to thank institute of health, Jimma University for funding the study. We also thank staffs of Jimma University Medical Center for providing preliminary data for the development of the proposal in this study. Finally, our gratitude goes to the participants of the study.”

We note that you have provided additional information within the Acknowledgements Section that is not currently declared in your Funding Statement. Please note that funding information should not appear in the Acknowledgments section or other areas of your manuscript. We will only publish funding information present in the Funding Statement section of the online submission form.

Please remove any funding-related text from the manuscript and let us know how you would like to update your Funding Statement. Currently, your Funding Statement reads as follows:

‘The authors received no specific funding for this work”

Please include your amended statements within your cover letter; we will change the online submission form on your behalf.

**Response: thank you very much for your comment. We have corrected it on the revised manuscript you can see it.**

**Question: #13.** Your ethics statement should only appear in the Methods section of your manuscript. If your ethics statement is written in any section besides the Methods, please move it to the Methods section and delete it from any other section. Please ensure that your ethics statement is included in your manuscript, as the ethics statement entered into the online submission form will not be published alongside your manuscript.

**Response: thank you for your remark. We have corrected it in the revised manuscript.**

**Question: #14.** We note that you have referenced (Bisanju WR et al. [4]) which has currently not yet been accepted for publication. Please remove this from your References and amend this to state in the body of your manuscript: (ie “Bisanju WR et al. [Unpublished]”) as detailed online in our guide for authors [http://joujuju8trnals.plos.org/plosone/s/submission-guidelines#loc-reference-style](http://journals.plos.org/plosone/s/submission-guidelines#loc-reference-style).

**Response: thank you for your comment. The paper is published on the journal of** *International Journal of Innovative Research in Medical Science (IJIRMS) with DOI:* [*https://doi.org/10.23958/ijirms/vol06-i12/1307*](https://doi.org/10.23958/ijirms/vol06-i12/1307)

**Question: #15.** Please include a separate caption for each figure in your manuscript

**Response: thank you for your remark. We have included it based on your request.**

**Question: #16.** Thank you for your attention to our requests, in the ethics statement we have noted that verbal consent was obtained from the parents or relatives of the minors included in the study. Please could you revise this statement clarify whether the relatives are also considered as legal guardians of the minors.

**Response:** **Sorry for the confusion we have created. In the context (Ethiopia) children are spent their time with their parents or relatives who are their legal guardians. During the data collation time we asked them whether they are their parents or relatives.**

**Response for reviewer**

**Question: #1.** In title, I believe the word ¨overall ¨is not important, why because we are measuring the time the patient spent from entry to exit which by default is the overall time spent by the patient.

**Response: thank you for your comment. You are right the term “entry to exit” can explain the paper but when the term “overall” includes in the paper it gives more power.**

**Question: #2.** . In Abstract, under background section, researchers should start with the definition of Overall time. its importance in relation to quality of care, and then describe the existing gaps that have led them to conduct this study.

**Response: thank you very much for your constructive comments. We have modified it on the revised manuscript you can see it.**

**Question: #3.** In conclusion section of the abstract, the authors stated that "The majority of the patients stayed for an extended period of time”. However, the extended time is not clearly operationalized in this document. The authors have found several numbers of minutes, but their implications are not included.

**Response:** thank you for your remark. “extended period of time” mean long overall time. We have operetionalized it on the method part of the revised manuscript.

**Question: #4**. This strongly relates to a high patient load, an absence of some services, being referred patients, and a lack of professional responsiveness". This has not been studied. The authors should interpret the findings of their study and put necessary recommendation based on what they have found. An absence of some services, and a lack of professional responsiveness were not shown to be associated in result section. there is over interpretation of the findings.

**Response: thank you for your insight. All those variables were assessed and became associated with the overall time except some of them associated only at bivariate analysis. (For more information you can see it in the revised manuscript as supporting information).**

**Question: #5.** In introduction section, the authors did not include the standard time that one patient should stay in health care facility. As a result, we cannot determine the implication of overall time spent by the patients. Whether they are below the standard or above the standard time?
This information is very important to forward the recommendation the included hospitals.

**Response:** thank you. The standards incorporate only the service time but this study may allows for the future to draw the standards that state the overall time that spent as an outpatient in the hospital because currently the Ethiopian hospitals are on reform.

**Question: #6**. The abbreviation should be written in expanded form when they are used for the first time in the text, thereafter the authors can use the abbreviation only. Eg. Outpatient Department (OPD), Jimma University Medical Center(JUMC).

**Response: thank you for your comment. We have corrected it on the revised manuscript.**

**Questions: #7.** The information in line 87-91 should be modified as below or the authors can consider another better alternative.
The length of time a patient spends in the hospital is one indicator of the service quality. In this regard, Jimma University developed a citizen charter in October 2016 to deliver timely services at Jimma University Medical Center (JUMC). However, the overall time that patients spent in JUMC from the time they enter to the time they leave the hospitals has not been studied. Therefore, this study aimed to evaluate the overall time patients spent from entry to exit and associated factors in out-patient departments of public hospitals of Jimma Zone.
But this works if the study setting is only JUMC .

**Response: thank you very much for your comment. We have modified it in the revised manuscript.**

**Question: #8.** In study setting section the authors should describe hospitals selected for this study in terms of their capacity, major service the hospitals are rendering, and the annual number patient follows up.

**Response: thank you for your constructive comment. We have incorporated in the revised manuscript.**

**Question: #9.** The issue of validity and reliability of instruments are not addressed and the source from which these instruments were adopted or adapted is not cited.

**Response: Thank you for your comment. We have incorporated your comments in the revised manuscript accordingly.
Question: #10.** . In line 155 ¨R2 ¨ should be R2.
**Response: thank you. We have corrected it in the revised version of the manuscript.**

**Question: #11.** The information in lines 156-158 should be described in dedicated result section and removed from method section.

**Response:** Thank you for your remark. We have modified it based on your request in the revised version of the manuscript.

**Question: #12.** In table 1: it is not clear why the number are different from 236 for some variables eg, educational status 172, monthly income 72, employment status 134.

**Response: thank you very much for your comment. Our study included both pediatric and adult age groups as study participants that means those pediatrics age groups create variation in educational status (children with age of below education unable to classify as “unable to read and write” rather we left them as its) and employment status (according to our country labor organization the minimum age to start work is 14 so individuals with age of below 14 left as its because we cannot said unemployment) . For income its below the employment status because for “house wife” and “unemployed” individuals there was no any monthly income.**

**Question: #13.** In table 2, pre-visit facility characteristics of respondents prior coming to the outpatient units,
This table is dedicated for pre visit facility characteristics; therefore, the table should be about those who had gone to other health care facilities(N=121) prior to coming to the outpatient units the selected hospitals. However, it is not clear why the authors are interested to include the type of visit and frequency of visit for all participants (n=236) in this table.

**Response: sorry for creating confusion on table 2. But we consider that the reason not visiting other health care facility was pre-visit characteristics and the remaining variables may go with the 3^rd^ table but the 3^rd^ table was bulky.**

**Question: #14.** It is not clear why the authors used different abbreviations for the same variables in different table. (Dics*** and Other Dxics***) in table 4 and 5, respectively

**Response: sorry for this editing error we have corrected it in the revised version of the manuscript.**

**Question: #15.** The waiting time, service time and overall time should be described based on the types of the hospitals selected for this study.

**Response: thank you for your constructive comment. We have modified it in the revised version of the manuscript and submitted as supporting information (you can see the revised version of the manuscript).**

**Question: #16.** The authors did not include the results of bivariate analysis.

**Response: thank you for your constructive comment. We have modified it in the revised version of the manuscript and submitted as supporting information (you can see the revised version of the manuscript).**

**Question: #17.** In the result section, the overall time was described in terms of the minutes, however, the discussion section the overall time is presented by hours. It should be consistent throughout sections.

**Response: thank you for your comment. We have set both in minutes and hours because some literatures set in hours so for easy comparisons we set in hours.**

**Question: #18.** In line 233" Indies" should be India

**Response: thank you for the comment. We have modified it in the revised version of the manuscript.**

**Question: #19.** . Information in lines 236-240 need to be cited.

**Response: thank you for the comment. We have modified it in the revised version of the manuscript.**

**Question: #20.** Even though the authors have included three hospitals, when they justify the observed differences, they used JUMC only, why the other included hospitals are ignored in the discussion? **Response: sorry for the confusion, since the majority of the sample (177)have been taken from JUMC (that is the largest teaching and referral hospital in southwest part of the Ethiopia). But know based on your comment we have tried to discuss based on the selected hospitals.**

**Question: #21.** In lines 255-259, did the authors assess those variables? professionals’ not arrive timely to the OPD?? Did you assess this?

**Response: thank you for your remark. As on variable we assessed about unit of delay then if “yes”, we have asked reason of delay (open ended) during this time the participant’s respond a multiple reasons. After that we have coded into major reasons and analyzed it. From those options “professionals not arrive timely” was one coded reason of delay.**

**Question: #22.** In line 317 it is not clear when to say long overall times as we do not have the standard in this document. This should have been operationalized in method section.

**Response: thank you for your valuable comment. We have operationalized it in the method part of the revised version of the manuscript.**

**Question: #23.** In this study authors used 3 hospitals that have significant difference in terms of the level of care, their type, number of patient flow, administration and etc. in this case it is not clear to which group of patients they want to generalize the results of their study.

**Response: thank you for the comment. The result was generalized based on the finding with comparison of the citizen charter stated time.**

**Question: #24.** Overall, the discussions are not adequate and the results are interpreted in terms of one institution (JUMC) ignoring the other two included hospitals.

Response: thank you, we have corrected it in the revised version of the manuscript.

**Question: #25.** . In abbreviation section
FNA/C, GOPD, and IOM should be added
**response: thank you for the comment. We have corrected it in the revised version of the manuscript.**

**Question: #26.** Some references are incomplete. Please thoroughly revise them. E.g, #20
**Response: thank you for your comment. We have corrected it in the revised version of the manuscript.**

**Question: #27.** Additionally, the manuscript has serious grammatical and language problems.

**Response**: **Sorry for the confusion we have created in the initial submission. Hence, we have tried to address all the problems concerning to the grammatical and language issues in the revised manuscript.**
