# Supplementary material for: Overall time spent by clients from entry to exit and associated factors in out-patient departments in public hospitals of Jimma Zone southwest, Ethiopia
Source: PLoS One. 2024 Mar 7;19(3):e0296630. doi: 10.1371/journal.pone.0296630 (PMC10919670; doi:10.1371/journal.pone.0296630)
Supplement: Attachment — Submitted filename: Response for Reviewers.docx [file pone.0296630.s008.docx]

Date: March 29, 2023

Manuscript ID: PONE-D-22-02050R1

Overall Time spent by clients from entry to exit and associated factors in out-patient departments in public hospitals of Jimma Zone Southwest, Ethiopia

Corresponding authors: Zebader Walle (MPh)

Dear Dr. Dragan Pamucar

PLOS ONE

Thank you for your letter dated January 30, 2023 with a decision of major revision needed. We were pleased to know that our manuscript was considered potentially acceptable for publication in PLoS ONE, subject to adequate revision as requested by the reviewers, academic editors and the journal requirements. Based on the instructions provided in your letter, we uploaded the file of the rebuttal letter; the marked up copy of the revised manuscript highlighting the changes made in the original submitted version and the clean copy of the revised manuscript.

We have revised all sections of the manuscript using the comments from the editors, independent reviewers, and the standard guideline of the journal. Hence, we have marked in red color all the changes made during the revision process. Beside this letter is our point-by-point response (rebuttal letter) to the comments made by the reviewers in the revised manuscript. We have agreed with almost all the comments and questions raised by the reviewers justification were provided for the disagreed comments and questions accordingly. We would like to take this opportunity to express our thanks to the reviewers for their valuable comments and to thank you for allowing us to resubmit a revision of the manuscript. The authors have declared that no competing interests exist.

I hope that the revised manuscript is accepted for publication in PLoS ONE.

Sincerely yours,

Zebader Walle (Debre Tabor University)

**Response for reviewer 1**

**Question: #1** I need more clarification on why some comments are not addressed. Therefore, I here with attached the same comments.

**Response: sorry for missing some comments in the previous revision. We have incorporated it in the revised version of manuscript.**

**Response to reviewer 2**

**Q#1:** Abstract: The sentences in the Conclusion and Recommendation section are still overstatements. Even though the data in the supplementary tables can support the sentences, the statements cannot be concluded by the contents of the abstract. I think that abstract should be independent and self-sufficient.

Response: thank you for your insight. We have incorporated it based on your request in the revised version of the manuscript.

**Q #2:** Results and Discussion: Although the authors addressed the previous Questions from the former reviewer, such as Questions #4 and #21, by adding the information as the supplementary tables, I think it is not enough. If the authors want to insist that "lack of professional responsiveness" or the specific character of the referral hospital (e.g., most physicians are interns) could be the main reasons for the long overall time, the authors should put the data in the main document rather than providing the data in the supplementary files.

**Response:** thank you for your suggestion. In the main document of table 3 there is the reason of delay (long overall time) in the hospitals. From the reasons “professionals not response timely or late” was the one reason. This may be happen because of the one selected hospital is referral and teaching that has morning session for intern students whom respond in most OPD of the assessment center.

**Q#3**: Table 6. Please provide the references for each variable as shown in the supplementary files.

**Response:** thank you for your comment we have corrected it in the revised version of the manuscript.
