# Supplementary material for: Overall time spent by clients from entry to exit and associated factors in out-patient departments in public hospitals of Jimma Zone southwest, Ethiopia
Source: PLoS One. 2024 Mar 7;19(3):e0296630. doi: 10.1371/journal.pone.0296630 (PMC10919670; doi:10.1371/journal.pone.0296630)
Supplement: Attachment — Submitted filename: reviewer comments to be sent to plosone.docx [file pone.0296630.s005.docx]

Date 12/07/2022
To Editorial Board,
PLOS ONE
REVIEW COMMENTS

TITLE: Overall Time spent by clients from entry to exit and associated factors in out-patient departments in public hospitals of Jimma Zone Southwest, Ethiopia

Manuscript number: PONE-D-22-02050

Thank you for inviting me to review this interesting article. Please kindly find the comments provided.

**Comments**

1. In title, I believe the word ¨overall ¨is not important, why because we are measuring the time the patient spent from entry to exit which by default is the overall time spent by the patient.
2. **In Abstract**, under background section, researchers should start with the definition of Overall time. its importance in relation to quality of care, and then describe the existing gaps that have led them to conduct this study.
3. In conclusion section of the abstract, the authors stated that "The majority of the patients stayed for an extended period of time”. However, the extended time is not clearly operationalized in this document. The authors have found several numbers of minutes, but their implications are not included.
4. .

*"This strongly relates to a high patient load, an absence of some services, being referred patients, and a lack of professional responsiveness".* This has not been studied. The authors should interpret the findings of their study and put necessary recommendation based on what they have found. An absence of some services, and a lack of professional responsiveness were not shown to be associated in result section. there is over interpretation of the findings.

5. **In introduction section**, the authors did not include the standard time that one patient should stay in health care facility. As a result, we cannot determine the implication of overall time spent by the patients. Whether they are below the standard or above the standard time?

This information is very important to forward the recommendation the included hospitals.

The abbreviation should be written in expanded form when they are used for the first time in the text, thereafter the authors can use the abbreviation only. Eg. Outpatient Department (OPD), Jimma University Medical Center(JUMC).

6. The information in line 87-91 should be modified as below or the authors can consider another better alternative.

The length of time a patient spends in the hospital is one indicator of the service quality. In this regard, Jimma University developed a citizen charter in October 2016 to deliver timely services at Jimma University Medical Center (JUMC). However, the overall time that patients spent in JUMC from the time they enter to the time they leave the hospitals has not been studied. Therefore, this study aimed to evaluate the overall time patients spent from entry to exit and associated factors in out-patient departments of public hospitals of Jimma Zone.

But this works if the study setting is only JUMC .

**Methods**

7. In study setting section the authors should describe hospitals selected for this study in terms of their capacity, major service the hospitals are rendering, and the annual number patient follows up.
8. The issue of validity and reliability of instruments are not addressed and the source from which these instruments were adopted or adapted is not cited.

**Result**

9. In line 155 ¨R2 ¨ should be R^2.^
10. The information in lines 156-158 should be described in dedicated result section and removed from method section.
11. In table 1: it is not clear why the number are different from 236 for some variables eg, educational status 172, monthly income 72, employment status 134.
12. In table 2, p*re-visit facility characteristics of respondents prior coming to the outpatient units,*

*This table is dedicated for pre visit facility characteristics; therefore, the table should be about those who had gone to other health care facilities(N=121) prior to coming to the outpatient units the selected hospitals. However, it is not clear why the authors are interested to include the type of visit and frequency of visit for all participants(n=236) in this table.*

13. *It is not clear why the authors used different abbreviations for the same variables in different table. (Dics*** and Other Dxics***) in table 4 and 5, respectively.*
14. *The waiting time, service time and overall time should be described based on the types of the hospitals selected for this study.*
15. *The authors did not include the results of bivariate analysis.*

*Discussion,*

16. *In the result section, the overall time was described in terms of the minutes, however, the discussion section the overall time is presented by hours. It should be consistent throughout sections.*
17. *In line 233*" Indies" should be India
18. Information in lines 236-240 need to be cited.
19. Even though the authors have included three hospitals, when they justify the observed differences, they used JUMC only, why the other included hospitals are ignored in the discussion?
20. In lines 255-259, did the authors assess those variables? professionals’ not arrive timely to the OPD?? Did you assess this?
21. In line 317 it is not clear when to say long overall times as we do not have the standard in this document. This should have been operationalized in method section.
22. *In this study authors used 3 hospitals that have significant difference in terms of the level of care, their type, number of patient flow, administration and etc. in this case it is not clear to which group of patients they want to generalize the results of their study.*
23. *Overall, the discussions are not adequate and the results are interpreted in terms of one institution (JUMC) ignoring the other two included hospitals.*
24. *In abbreviation section*

FNA/C, GOPD, and IOM should be added

References

25. Some references are incomplete. Please thoroughly revise them. eg,. #20
26. Additionally, the manuscript has serious grammatical and language problems.
